# Supplementary material for: B-1a cells acquire their unique characteristics by bypassing the pre-BCR selection stage
Source: Nat Commun. 2019 Oct 18;10:4768. doi: 10.1038/s41467-019-12824-z (PMC6802180; doi:10.1038/s41467-019-12824-z)
Supplement: Supplementary file 1 — Supplementary Information [file 41467_2019_12824_MOESM1_ESM.pdf]

B-1a cells acquire their unique characteristics by bypassing the pre-BCR selection stage.

Wong et al.

Supplementary Tables 1-2

		No γ-H2AX association number	%	γ-H2AX association number	%	Sample size (cells)
Exp1	BM pro-B	123	96.9	4	3.1	127
	BM pre-B	86	72.9	32	27.1	118
	FL pro-B	125	84.5	23	15.5	148
Exp2	BM pro-B	231	97.1	7	2.9	238
	BM pre-B	139	67.1	68	32.9	207
	FL pro-B	60	82.2	13	17.8	73
**TOTAL:**	**BM pro-B**	**354**	**97.0**	**11**	**3.0**	**365**
	**BM pre-B**	**225**	**69.2**	**100**	**30.8**	**325**
	**FL pro-B**	**185**	**83.7**	**36**	**16.3**	**221**
Statistical analysis (Fisher's exact test):				p-value		Level
BM pro-B vs BM pre-B				2.12e-15		***
BM pro-B vs FL pro-B				5.89e-6		***
BM pre-B vs FL pre-B				1.19e-3		**

Supplementary Table 1 Data table summarizing two independent experiments immuno-FISH experiments for γ-H2AX association with the *Igk* locus in bone marrow pro-B cells, bone marrow pre-B cells and Fetal liver pro-B cells. Significance level was determined using Fisher's exact test on the combined data from the two experiments.

Rearrangement sequencing primers	
Name	Primer Sequence 5’ to 3’
IgH V-FR3-1	AAGTTCAAGGGCAAGGCC
IgH V-FR3-2	CTCCAAGAGCCAAGTTTTCTT
IgH V-FR3-3	CAATCTCCAAGGATACCTCCA
IgH V-FR3-4	CGITTCACCATCTCCAGAGA
IgH V-12	ACTTTCTACAACCCATCTCT
IgH V-11	ACGCACCATCCATAAAGGAT
IgH V-6	AGGTTCACCATCTCAAGAGA
IgH V-9	TTCAAGGGACGGTTTGCCTT
IgH reverse [J1/4]	CTTACCTGAGGAGACGGTGAC
IgH reverse [J2]	AGGACTCACCTGAGGAGAC
IgH reverse [J3]	AGGACTCACCTGCAGAGAC
Igk forward Vk -sense IgL	GGCTGCAGSTTCAGTGGCAGTGGRTCWGGRAC
Igk forward Vk9-128	TCAAGGTTCAGTGGCAGTGGGTCTTAGTC
Igk forward Vk14-126	GGGGTCCCATCAAGATTCAGTGGCAGTGGATC
Igk reverse [J1/2]	TACTTACGTTTKATTTCCAGCTTGGT
Igk reverse [J3/4]	TACTTAGGTTTTATTTCCAGTCTGGT
Igk reverse [J5]	TACTTACGTTTCAGCTCCAGCTTGGT

Supplementary Table 2 List of primers used to PCR amplify *Igh* and *Igk* DNA rearrangements for B cell repertoire sequencing.
