# Supplementary material for: B-1a cells acquire their unique characteristics by bypassing the pre-BCR selection stage
Source: Nat Commun. 2019 Oct 18;10:4768. doi: 10.1038/s41467-019-12824-z (PMC6802180; doi:10.1038/s41467-019-12824-z)
Supplement: Supplementary file 3 — Reporting Summary [file 41467_2019_12824_MOESM3_ESM.pdf]

nature research

Corresponding author(s): Jane Skok

Last updated by author(s): Sep 20, 2019

Reporting Summary

Nature Research wishes to improve the reproducibility of the work that we publish. This form provides structure for consistency and transparency in reporting. For further information on Nature Research policies, see Authors & Referees and the Editorial Policy Checklist.

Statistics

For all statistical analyses, confirm that the following items are present in the figure legend, table legend, main text, or Methods section.

n/a	Confirmed	
☐	☒	The exact sample size (n) for each experimental group/condition, given as a discrete number and unit of measurement
☐	☒	A statement on whether measurements were taken from distinct samples or whether the same sample was measured repeatedly
☐	☒	The statistical test(s) used AND whether they are one- or two-sided<br>*Only common tests should be described solely by name; describe more complex techniques in the Methods section.*
☒	☐	A description of all covariates tested
☒	☐	A description of any assumptions or corrections, such as tests of normality and adjustment for multiple comparisons
☐	☒	A full description of the statistical parameters including central tendency (e.g. means) or other basic estimates (e.g. regression coefficient) AND variation (e.g. standard deviation) or associated estimates of uncertainty (e.g. confidence intervals)
☐	☒	For null hypothesis testing, the test statistic (e.g. F, t, r) with confidence intervals, effect sizes, degrees of freedom and P value noted<br>*Give P values as exact values whenever suitable.*
☒	☐	For Bayesian analysis, information on the choice of priors and Markov chain Monte Carlo settings
☒	☐	For hierarchical and complex designs, identification of the appropriate level for tests and full reporting of outcomes
☒	☐	Estimates of effect sizes (e.g. Cohen's d, Pearson's r), indicating how they were calculated

Our web collection on statistics for biologists contains articles on many of the points above.

Software and code

Policy information about availability of computer code

Data collection	no software was used
Data analysis	no software was used

For manuscripts utilizing custom algorithms or software that are central to the research but not yet described in published literature, software must be made available to editors/reviewers. We strongly encourage code deposition in a community repository (e.g. GitHub). See the Nature Research guidelines for submitting code & software for further information.

Data

Policy information about availability of data

All manuscripts must include a data availability statement. This statement should provide the following information, where applicable:

- Accession codes, unique identifiers, or web links for publicly available datasets
- A list of figures that have associated raw data
- A description of any restrictions on data availability

Data that support findings of this study have been deposited in the Gene Expression Omnibus (GEO) database under the accession code GSE104111, [https://www.ncbi.nlm.nih.gov/geo/query/acc.cgi?acc=GSE104111].

Field-specific reporting

Please select the one below that is the best fit for your research. If you are not sure, read the appropriate sections before making your selection.

☒ Life sciences ☐ Behavioural & social sciences ☐ Ecological, evolutionary & environmental sciences

Life sciences study design

All studies must disclose on these points even when the disclosure is negative.

Sample size	No sample size calculation was used. Samples sizes were determined based on when the data maintained statistical significance as well as ensured that the number of mice used were not in excess. Similar results from multiple experiments gave us confidence in our results and their claims as this lessens the chances for batch effects.
Data exclusions	no data was excluded.
Replication	All attempts at replication were successful.
Randomization	Mice were used based on genotype irregardless of other potential biases, litter mates were used when possible.
Blinding	For FISH and Immuno-FISH scoring was done blinded to avoid scoring biases. For Repertoire sequencing, samples during library prep were coded to prohibit sample handling biases.

Reporting for specific materials, systems and methods

We require information from authors about some types of materials, experimental systems and methods used in many studies. Here, indicate whether each material, system or method listed is relevant to your study. If you are not sure if a list item applies to your research, read the appropriate section before selecting a response.

Materials & experimental systems

n/a	Involved in the study	
☐	☒	Antibodies
☒	☐	Eukaryotic cell lines
☒	☐	Palaeontology
☐	☒	Animals and other organisms
☒	☐	Human research participants
☒	☐	Clinical data

Methods

n/a	Involved in the study	
☒	☐	ChIP-seq
☐	☒	Flow cytometry
☒	☐	MRI-based neuroimaging

Antibodies

Antibodies used

Antibody	Company	Cat. #	Clone #	Lot #:	Manufacturer validated
Anti- CD45R(B220)	BD	553092	RA3-6B2	4073804	YES
Anti- CD19	BD	557655	1D3	24999	YES
Anti- IgMb	BD	553520	AF6-78	2202585	YES
Anti- IgM	BD	553437	II/41	64277	YES
Anti- IgM	BD	743324	II/41	7290774	YES
Anti- IgK	BD	562476	187.1	7116990	YES
Anti- IgK	BD	562888	187.1	3249768	YES
Anti- CD117(cKit)	BD	553356	2B8	4084660	YES
Anti- CD2	BD	553111	RM2-5	33590	YES
Anti- CD25	BD	553866	PC61	27378	YES
Anti-CD90.2	eBioscience	25-0902-82	3-2.1	E07588-1630	YES
anti-ENPP1(PC1)	eBioscience	149207	YE1/19.1	n/a	YES
Anti-CD93	BD	561990	AA4.1	n/a	YES
Anti- CD5	BD	553022	53-7.3	4080687	YES
Anti-TCRB	eBioscience	47-5961-82	H57-597	E08478-1643	YES
Anti-CD8a	BD	553031	53-6.7	36536	YES
Anti-CD4	BD	553051	RM4-5	3304739	YES
Anti-VH12	custom	n/a	5C5	n/a	Arnold et. al. (Below)

Arnold, B. L. W. et al. Development of B-1 Cells: Segregation of Phosphatidyl Choline-specific B Cells to the B-1 Population Occurs After Immunoglobulin Gene Expression. 179, 1585–1595 (1994).

Validation

All antibodies were validated by the manufacturers

Animals and other organisms

Policy information about studies involving animals; ARRIVE guidelines recommended for reporting animal research

Laboratory animals

Fetal liver B cells were extracted from fetuses E16.5-17.5
BM pro-B and BM pre-B cells were extracted from 5-6 week old mice from femurs and tibias/fibulas

Wild-type lab mice were from Taconic (C57BL/6NTac)
Igll1-/- mice were from Jackson laboratory (Stock Number: 002401)
CaStat5 mice used as in: Burchill, M. a et al. Distinct effects of STAT5 activation on CD4+ and CD8+ T cell homeostasis: development of CD4+CD25+ regulatory T cells versus CD8+ memory T cells. J. Immunol. 171, 5853–64 (2003).

Wild animals	The study did not involve wild animals.
Field-collected samples	The study did not involve samples collected from the field.
Ethics oversight	IACUC IA15-01468

Note that full information on the approval of the study protocol must also be provided in the manuscript.

Flow Cytometry

Plots

Confirm that:

☒ The axis labels state the marker and fluorochrome used (e.g. CD4-FITC).

☒ The axis scales are clearly visible. Include numbers along axes only for bottom left plot of group (a 'group' is an analysis of identical markers).

☒ All plots are contour plots with outliers or pseudocolor plots.

☒ A numerical value for number of cells or percentage (with statistics) is provided.

Methodology

Sample preparation	Biological sources included Spleens,
Instrument	for sorting: BD FACSAria IIu SORP For analysis: BD LSRII
Software	Flow Cytometry data was collected using FACS DIVA and analyzed using Flowjo
Cell population abundance	Cell populations were always at very high purity >95%
Gating strategy	During preliminary gating, Small sized fragments were gated out to eliminate cell debris, then two single cell gates were created to eliminate doublets. first FSC-A vs FSC-H followed by SSC-A vs SSC-H.

☒ Tick this box to confirm that a figure exemplifying the gating strategy is provided in the Supplementary Information.
